# The Practitioners' Bookshelf

**Published:** 1892-12-17

**Authors:** 


### THE PRACTITIONERS' BOOKSHELF.

### THE HUMAN BODY.

A really excellent little book* has been added to the number of elementary text books. No amount of letter-press ever conveyed to the young idea the same vivid comprehension of a subject as some pictorial method. In this little work, most aptly entitled "The Human Body," Mr. Lancaster has achieved all that human ingenuity could devise to represent within the cover of a book the exact position and arrangement of the various organs and their tributaries by the means of the "mannikin" accompanying the book. This little lay figure enfolds within the bony structure of the body, as represented on the upmost folds of the paper model, the complex human system, arranged layer above layer, each fold showing the outer and inner appearance of the organ, carefully depicted. The student learns in this manner, as from the flat it would be impossible otherwise to learn, the position and connection of all parts of the body. The book contains letterpress of the simplest. Facts are stated in a brief but most comprehensive manner. Appended to the mannikin is a complete and numbered description of all its component parts. Where elementary physiology is taught, and to the hospital probationer, the little book will be invaluable.

### A NEW PERIODICAL.

A new monthly, the *Medical Magazine*, has appeared in our midst, and although it is true that of the making of periodicals, as well as books, there is no end, there are certain productions which so clearly fill a space, that they are welcomed, and they flourish, large as the literary family may be. That the *Medical Magazine* is one of these we may venture to predict. It does not so much profess to instruct as to philosophise. It should afford a field for those who, having learnt and practised the technicalities of medical science, have formed, as the result of their experience, views of wider application and interest than can be expressed in the purely technical medical periodicals previously existing. In the copy of the magazine now before us, that for the month of October, we have briefly placed before us the present practical application of sanitary science. The article entitled "The Victorian Era: the Age of Sanitation," is by Sir Charles Cameron, who, short as is the space allotted to so comprehensive a subject, has managed to supply us with a complete and critical history of it, which will prove both interesting and useful as a bird's-eye view on sanitation. Many golden words of wisdom are uttered in the addresses of our leading medical men at the opening of a session at the schools. The occasion is one in which many men deliver to their hearers the maxims formed on the experience of a life of useful work. The best of these addresses will evidently find a welcome within the pages of the new magazine, to the benefit of its readers. There is also a Reviewing corner and one for Editorial Notes. We shall look forward with pleasure to the progress and development of this the latest medical periodical.

### LETTS' DIARIES.

Letts' Diaries are as well-known as Pears' Soap or Cadbury's Cocoa; and for the same reason, that they are well worth their money. If one asks, who is Letts? the answer is that at present he is Cassell and Co., and if there be anybody more famous than Letts for the production of the very kind of books that the majority of people want, that somebody is Cassell and Co. Sometimes a mere catalogue of names is quite interesting reading. Who would think, for example, that so many different diaries and similar publications as we give here could by any possibility find sufficient buyers to make their production possible? There is this "Doctors'" Diary we hold in our hand, the "Clerical" Diary, the "Universal," the "Scribbling," the "Tablet," the "Rough," and a host of others of the diary breed. As variants we have the "Church and Chapel Register," the "Letter Book," the "Game Book," the "Housekeeper's," "Poultry Keeper's," "Stable Keeper's," and no end of others. "Captain Cuttle's Index Book" must be a very amusing production. What it contains we are at a loss to guess, unless it be a full description of handbooks and wooden legs, and other things which, when found, it is desirable to make a note of. Letts, indeed, is a perennial friend. We associate him with Christmas and the end of the year. To many of us he is our first Christmas present, and we welcome him accordingly. Those medical men who are not fortunate enough to have already received the Doctor's Diary as a present, may at once buy him in the full confidence that they will never repent of their bargain.

* "The Human Body." Illustrated by Diagrams and a Coloured Mannikin. By Owen Lankester, M.R.C.S. (Allman and Sons.)

